# 2,2′-Bis(9-hydr­oxy-9-fluoren­yl)biphen­yl–ethyl acetate (1/1)

**DOI:** 10.1107/S1600536808023271

**Published:** 2008-07-31

**Authors:** Lidiya Izotova, Jamshid Ashurov, Bakhtiyar Ibragimov, Edwin Weber

**Affiliations:** a Institute of Bioorganic Chemistry, Academy of Sciences of Uzbekistan, H. Abdullaev 83, Tashkent 100125, Uzbekistan; b Institut für Organische Chemie, TU Bergakademie Freiberg, Leipziger Strasse 29, D-09596 Freiberg/Sachsen, Germany

## Abstract

In the title host–guest compound, C_38_H_26_O_2_·C_4_H_8_O_2_, the ethyl acetate mol­ecule (guest), which adopts a fully extended conformation, and the biphenyl derivative (host) are connected via O—H⋯O hydrogen bonds [H⋯O = 1.90 (3) Å] into discrete assemblies. The hydro­carbon skeleton of the host mol­ecule deviates only slightly from C_2_ symmetry. The OH groups of the host are involved in intra­molecular O—H⋯O hydrogen bonding [H⋯O = 1.83 (3) Å].

## Related literature

For related literature, see: Barbour *et al.* (1993 ▶); Ibragimov *et al.* (2001 ▶); Sardone (1996 ▶); Sumarna *et al.* (2003 ▶); Weber *et al.* (1993 ▶).
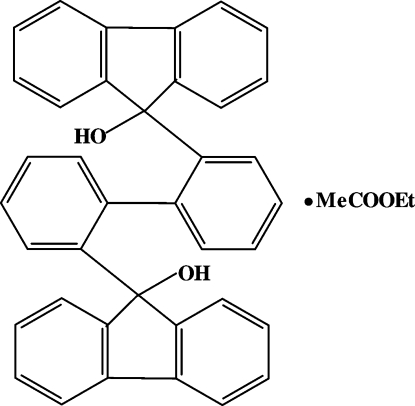


## Experimental

### 

#### Crystal data

C_38_H_26_O_2_·C_4_H_8_O_2_
*M* *_r_* = 602.69
Monoclinic, 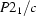

*a* = 11.645 (2) Å
*b* = 16.364 (3) Å
*c* = 17.471 (3) Å
β = 97.72 (3)°
*V* = 3299.1 (10) Å^3^
*Z* = 4
Mo *K*α radiation
μ = 0.08 mm^−1^
*T* = 293 (2) K
0.4 × 0.2 × 0.2 mm

#### Data collection

Stoe STADI4 diffractometer
Absorption correction: none
5807 measured reflections
5650 independent reflections
3654 reflections with *I* > 2σ(*I*)
3 standard reflections every 100 reflections intensity decay: 2.6%

#### Refinement

*R*[*F* ^2^ > 2σ(*F* ^2^)] = 0.068
*wR*(*F* ^2^) = 0.141
*S* = 1.20
5650 reflections
424 parameters
H atoms treated by a mixture of independent and constrained refinement
Δρ_max_ = 0.24 e Å^−3^
Δρ_min_ = −0.16 e Å^−3^

### 

Data collection: *STADI4* (Stoe & Cie, 1997 ▶); cell refinement: *STADI4*; data reduction: *X-RED* (Stoe & Cie, 1997 ▶); program(s) used to solve structure: *SHELXS97* (Sheldrick, 2008 ▶); program(s) used to refine structure: *SHELXL97* (Sheldrick, 2008 ▶); molecular graphics: *XP* (Siemens, 1994 ▶); software used to prepare material for publication: *SHELXL97*.

## Supplementary Material

Crystal structure: contains datablocks I, global. DOI: 10.1107/S1600536808023271/gk2155sup1.cif

Structure factors: contains datablocks I. DOI: 10.1107/S1600536808023271/gk2155Isup2.hkl

Additional supplementary materials:  crystallographic information; 3D view; checkCIF report

## Figures and Tables

**Table 1:** Hydrogen-bond geometry (Å, °)

*D*—H⋯*A*	*D*—H	H⋯*A*	*D*⋯*A*	*D*—H⋯*A*
O2—H1⋯O3^i^	0.88 (4)	1.90 (4)	2.779 (3)	173 (4)
O1—H2⋯O2	0.93 (4)	1.84 (4)	2.739 (3)	161 (3)

Symmetry code: (i) 
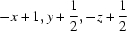
.

**Fig. 1.**
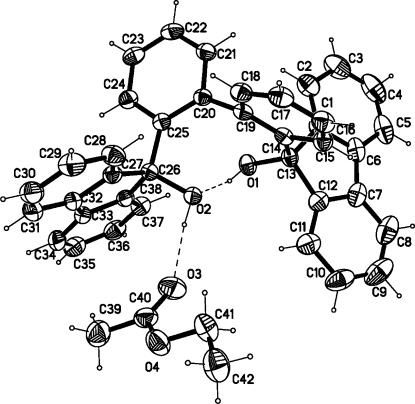
Perspective view of the title compound, showing 30% probability displacement ellipsoids for the non-H atoms. Dashed lines represent hydrogen bonds.

**Fig. 2.**
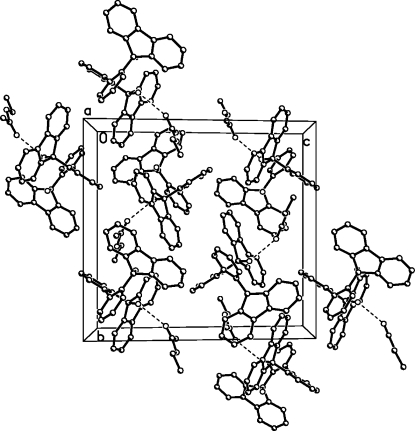
Packing diagram of the title compound (I) viewed down the a axis. H atoms have been ommited for clarity. Hydrogen bonds are shown as dashed lines.

## supplementary crystallographic information

### Comment

Crystalline inclusion compounds (clathrates, host–guest complexes) are of increasing importance in supramolecular chemistry because of their significant potential in addressing a variety of fundamental and practical issues. 2,2'-Bis(9-hydroxy-9-fluorenyl)biphenyl (I) is a host compound with good clathrate-forming ability and the crystal structures of its inclusion compounds with acetonitrile, cyclohexanone, n-propylamine (Barbour *et al.*, 1993), acetone (three solvates) (Sardone, 1996; Ibragimov *et al.*, 2001) and chloroform (two solvates) (Sumarna *et al.*, 2003) were reported. Here, we report the crystal structure of a host–guest complex of (I) with ethyl acetate which resembles closely that of (I) with acetone (1/1) (Sardone, 1996). The molecule of (I) has three conformational degrees of freedom (rotation around the central aryl–aryl single bond and rotations around the aryl–fluorenyl bonds), however it exhibits considerable conformational rigidity due to the stabilizing effect of the intramolecular O—H···O hydrogen bond between the hydroxyl groups (Fig. 1, Table 1). The crystal packing is mainly stabilized by van der Waals forces (Fig.2).

### Experimental

2,2'-Bis(9-hydroxy-9-fluorenyl)biphenyl was synthesized according to the procedure described by Weber *et al.*, (1993). The stable in the air crystals were grown by slow evaporation from ethyl acetate solution.

### Refinement

H atoms from the OH groups were located from difference Fourier maps and fully refined. The remaining H atoms were positioned geometrically (C—H 0.93–0.98 Å) and refined as riding on their carrier atoms with *U*_iso_(H) = 1.2*U*_eq_(C), except the methyl groups where *U*_iso_(H) = 1.5*U*_eq_(C).

### Crystal data

**Table:**

C_38_H_26_O_2_·C_4_H_8_O_2_	*F*_000_ = 1272
*M**_r_* = 602.69	*D*_x_ = 1.213 Mg m^−^^3^
Monoclinic, *P*2_1_/*c*	Mo *K*α radiation λ = 0.71073 Å
Hall symbol: -P 2ybc	Cell parameters from 25 reflections
*a* = 11.645 (2) Å	θ = 10–20º
*b* = 16.364 (3) Å	µ = 0.08 mm^−^^1^
*c* = 17.471 (3) Å	*T* = 293 (2) K
β = 97.72 (3)º	Block, colourless
*V* = 3299.1 (10) Å^3^	0.4 × 0.2 × 0.2 mm
*Z* = 4	

### Data collection

**Table:**

Stoe STADI4 diffractometer	*R*_int_ = 0.0000
Radiation source: fine-focus sealed tube	θ_max_ = 25.0º
Monochromator: graphite	θ_min_ = 2.6º
*T* = 293(2) K	*h* = −13→11
ω/2θ scans	*k* = 0→19
Absorption correction: none	*l* = 0→20
5650 measured reflections	3 standard reflections
5807 independent reflections	every 100 reflections
3654 reflections with *I* > 2σ(*I*)	intensity decay: 2.6%

### Refinement

**Table:**

Refinement on *F*^2^	Hydrogen site location: inferred from neighbouring sites
Least-squares matrix: full	H atoms treated by a mixture of independent and constrained refinement
*R*[*F*^2^ > 2σ(*F*^2^)] = 0.068	*w* = 1/[σ^2^(*F*_o_^2^) + (0.0281*P*)^2^ + 2.047*P*] where *P* = (*F*_o_^2^ + 2*F*_c_^2^)/3
*wR*(*F*^2^) = 0.141	(Δ/σ)_max_ < 0.001
*S* = 1.20	Δρ_max_ = 0.24 e Å^−^^3^
5650 reflections	Δρ_min_ = −0.16 e Å^−^^3^
424 parameters	Extinction correction: SHELXL97 (Sheldrick, 2008), Fc^*^=kFc[1+0.001xFc^2^λ^3^/sin(2θ)]^-1/4^
Primary atom site location: structure-invariant direct methods	Extinction coefficient: 0.0048 (4)
Secondary atom site location: difference Fourier map	

### Special details

**Table:**

Geometry. All e.s.d.'s (except the e.s.d. in the dihedral angle between two l.s. planes) are estimated using the full covariance matrix. The cell e.s.d.'s are taken into account individually in the estimation of e.s.d.'s in distances, angles and torsion angles; correlations between e.s.d.'s in cell parameters are only used when they are defined by crystal symmetry. An approximate (isotropic) treatment of cell e.s.d.'s is used for estimating e.s.d.'s involving l.s. planes.
Refinement. Refinement of *F*^2^ against ALL reflections. The weighted *R*-factor *wR* and goodness of fit *S* are based on *F*^2^, conventional *R*-factors *R* are based on *F*, with *F* set to zero for negative *F*^2^. The threshold expression of *F*^2^ > σ(*F*^2^) is used only for calculating *R*-factors(gt) *etc*. and is not relevant to the choice of reflections for refinement. *R*-factors based on *F*^2^ are statistically about twice as large as those based on *F*, and *R*- factors based on ALL data will be even larger.

### Fractional atomic coordinates and isotropic or equivalent isotropic displacement parameters (Å^2^)

**Table:**

	*x*	*y*	*z*	*U*_iso_*/*U*_eq_	
O1	0.70308 (18)	0.69051 (14)	0.20949 (13)	0.0518 (6)	
O2	0.63649 (17)	0.85042 (13)	0.21819 (12)	0.0470 (5)	
C1	0.8676 (3)	0.62161 (19)	0.1739 (2)	0.0511 (8)	
C2	0.8325 (3)	0.5837 (2)	0.1044 (2)	0.0716 (11)	
H2A	0.7765	0.6073	0.0681	0.086*	
C3	0.8827 (4)	0.5090 (3)	0.0897 (3)	0.0937 (14)	
H3A	0.8596	0.4822	0.0432	0.112*	
C4	0.9657 (5)	0.4747 (3)	0.1430 (4)	0.0995 (17)	
H4A	0.9982	0.4248	0.1321	0.119*	
C5	1.0019 (3)	0.5126 (3)	0.2122 (3)	0.0828 (13)	
H5A	1.0588	0.4889	0.2478	0.099*	
C6	0.9523 (3)	0.5867 (2)	0.2280 (2)	0.0616 (10)	
C7	0.9683 (3)	0.6387 (2)	0.2964 (2)	0.0633 (10)	
C8	1.0404 (4)	0.6306 (3)	0.3666 (3)	0.0891 (14)	
H8A	1.0925	0.5874	0.3749	0.107*	
C9	1.0330 (5)	0.6877 (4)	0.4232 (3)	0.1026 (18)	
H9A	1.0814	0.6831	0.4698	0.123*	
C10	0.9559 (4)	0.7516 (3)	0.4126 (2)	0.0903 (14)	
H10A	0.9510	0.7885	0.4526	0.108*	
C11	0.8848 (3)	0.7614 (2)	0.3424 (2)	0.0701 (10)	
H11A	0.8334	0.8050	0.3343	0.084*	
C12	0.8930 (3)	0.7041 (2)	0.28501 (19)	0.0545 (9)	
C13	0.8229 (3)	0.70131 (18)	0.20394 (17)	0.0460 (7)	
C14	0.8464 (3)	0.77519 (18)	0.15426 (17)	0.0434 (7)	
C15	0.9585 (3)	0.8074 (2)	0.16490 (18)	0.0527 (8)	
H15A	1.0139	0.7835	0.2013	0.063*	
C16	0.9899 (3)	0.8735 (2)	0.1234 (2)	0.0592 (9)	
H16A	1.0655	0.8931	0.1315	0.071*	
C17	0.9087 (3)	0.9100 (2)	0.07009 (19)	0.0597 (9)	
H17A	0.9284	0.9551	0.0422	0.072*	
C18	0.7978 (3)	0.87899 (19)	0.05842 (18)	0.0524 (8)	
H18A	0.7434	0.9038	0.0219	0.063*	
C19	0.7638 (3)	0.81209 (18)	0.09903 (16)	0.0433 (7)	
C20	0.6441 (3)	0.77988 (17)	0.06964 (16)	0.0423 (7)	
C21	0.6384 (3)	0.73514 (19)	0.00138 (17)	0.0523 (8)	
H21A	0.7072	0.7234	−0.0180	0.063*	
C22	0.5355 (3)	0.7074 (2)	−0.03899 (18)	0.0584 (9)	
H22A	0.5351	0.6771	−0.0840	0.070*	
C23	0.4340 (3)	0.7258 (2)	−0.01101 (18)	0.0578 (9)	
H23A	0.3637	0.7078	−0.0371	0.069*	
C24	0.4363 (3)	0.77059 (19)	0.05528 (18)	0.0514 (8)	
H24A	0.3665	0.7831	0.0730	0.062*	
C25	0.5396 (3)	0.79833 (17)	0.09746 (16)	0.0414 (7)	
C26	0.5264 (2)	0.84892 (18)	0.16933 (16)	0.0424 (7)	
C27	0.4832 (3)	0.93557 (18)	0.14954 (17)	0.0470 (8)	
C28	0.5316 (3)	0.9954 (2)	0.10912 (19)	0.0614 (9)	
H28A	0.5993	0.9855	0.0878	0.074*	
C29	0.4774 (4)	1.0712 (2)	0.1008 (2)	0.0726 (11)	
H29A	0.5088	1.1124	0.0733	0.087*	
C30	0.3779 (4)	1.0858 (2)	0.1327 (2)	0.0762 (12)	
H30A	0.3433	1.1370	0.1270	0.091*	
C31	0.3284 (3)	1.0264 (2)	0.1728 (2)	0.0682 (10)	
H31A	0.2605	1.0367	0.1938	0.082*	
C32	0.3816 (3)	0.9507 (2)	0.18140 (17)	0.0511 (8)	
C33	0.3506 (3)	0.8771 (2)	0.22174 (17)	0.0503 (8)	
C34	0.2593 (3)	0.8615 (3)	0.2634 (2)	0.0678 (11)	
H34A	0.2048	0.9018	0.2692	0.081*	
C35	0.2510 (3)	0.7854 (3)	0.2959 (2)	0.0734 (12)	
H35A	0.1899	0.7743	0.3235	0.088*	
C36	0.3315 (3)	0.7256 (3)	0.28821 (19)	0.0673 (10)	
H36A	0.3241	0.6746	0.3105	0.081*	
C37	0.4236 (3)	0.7407 (2)	0.24757 (18)	0.0548 (9)	
H37A	0.4785	0.7005	0.2425	0.066*	
C38	0.4321 (3)	0.81679 (19)	0.21478 (16)	0.0451 (8)	
C39	0.5324 (3)	0.5281 (3)	0.1162 (2)	0.0869 (13)	
H39A	0.5772	0.4805	0.1328	0.130*	
H39B	0.5655	0.5751	0.1437	0.130*	
H39C	0.5330	0.5360	0.0618	0.130*	
C40	0.4118 (4)	0.5169 (2)	0.1319 (2)	0.0696 (11)	
C41	0.2259 (3)	0.5774 (3)	0.1255 (3)	0.0903 (13)	
H41A	0.2232	0.5760	0.1807	0.108*	
H41B	0.1883	0.5287	0.1026	0.108*	
C42	0.1664 (4)	0.6520 (3)	0.0910 (3)	0.0979 (15)	
H42A	0.0869	0.6516	0.1003	0.147*	
H42B	0.1695	0.6527	0.0364	0.147*	
H42C	0.2043	0.6997	0.1142	0.147*	
O3	0.3748 (3)	0.45679 (18)	0.15903 (17)	0.0996 (10)	
O4	0.3455 (2)	0.58069 (16)	0.11015 (16)	0.0795 (8)	
H2	0.677 (3)	0.741 (2)	0.223 (2)	0.092 (14)*	
H1	0.627 (3)	0.883 (2)	0.257 (2)	0.098 (15)*	

### Atomic displacement parameters (Å^2^)

**Table:**

	*U*^11^	*U*^22^	*U*^33^	*U*^12^	*U*^13^	*U*^23^
O1	0.0430 (13)	0.0475 (14)	0.0667 (15)	−0.0008 (11)	0.0142 (11)	0.0074 (12)
O2	0.0447 (13)	0.0490 (13)	0.0467 (13)	0.0032 (10)	0.0036 (10)	−0.0076 (11)
C1	0.0485 (19)	0.0426 (19)	0.065 (2)	−0.0026 (16)	0.0176 (17)	0.0049 (17)
C2	0.082 (3)	0.051 (2)	0.084 (3)	0.002 (2)	0.018 (2)	−0.008 (2)
C3	0.119 (4)	0.057 (3)	0.112 (4)	0.005 (3)	0.040 (3)	−0.018 (3)
C4	0.107 (4)	0.050 (3)	0.155 (5)	0.011 (3)	0.068 (4)	0.007 (3)
C5	0.063 (3)	0.059 (3)	0.132 (4)	0.013 (2)	0.037 (3)	0.035 (3)
C6	0.050 (2)	0.048 (2)	0.089 (3)	0.0038 (17)	0.021 (2)	0.021 (2)
C7	0.049 (2)	0.066 (2)	0.074 (3)	−0.0098 (19)	0.0057 (19)	0.030 (2)
C8	0.071 (3)	0.097 (4)	0.094 (3)	−0.016 (3)	−0.007 (3)	0.050 (3)
C9	0.104 (4)	0.126 (5)	0.071 (3)	−0.048 (4)	−0.016 (3)	0.042 (3)
C10	0.099 (4)	0.116 (4)	0.054 (3)	−0.047 (3)	0.007 (2)	0.004 (3)
C11	0.071 (3)	0.079 (3)	0.061 (2)	−0.021 (2)	0.011 (2)	0.002 (2)
C12	0.053 (2)	0.057 (2)	0.053 (2)	−0.0129 (18)	0.0085 (16)	0.0087 (17)
C13	0.0390 (18)	0.0460 (18)	0.0540 (19)	−0.0014 (14)	0.0100 (14)	0.0024 (15)
C14	0.0471 (19)	0.0391 (17)	0.0466 (18)	−0.0027 (14)	0.0155 (15)	−0.0051 (14)
C15	0.047 (2)	0.056 (2)	0.055 (2)	−0.0029 (17)	0.0090 (16)	−0.0008 (17)
C16	0.056 (2)	0.063 (2)	0.063 (2)	−0.0176 (18)	0.0205 (18)	−0.0044 (19)
C17	0.073 (3)	0.053 (2)	0.056 (2)	−0.0111 (19)	0.0217 (19)	0.0012 (17)
C18	0.061 (2)	0.048 (2)	0.0491 (19)	−0.0008 (17)	0.0138 (16)	0.0026 (16)
C19	0.0537 (19)	0.0388 (17)	0.0392 (16)	0.0013 (15)	0.0131 (14)	−0.0020 (14)
C20	0.0490 (19)	0.0391 (17)	0.0389 (16)	0.0011 (14)	0.0066 (14)	0.0019 (13)
C21	0.063 (2)	0.050 (2)	0.0453 (18)	0.0062 (17)	0.0116 (16)	−0.0004 (15)
C22	0.083 (3)	0.051 (2)	0.0395 (18)	−0.0038 (19)	0.0034 (18)	−0.0082 (16)
C23	0.066 (2)	0.059 (2)	0.0458 (19)	−0.0139 (18)	−0.0034 (17)	−0.0015 (17)
C24	0.051 (2)	0.054 (2)	0.0489 (19)	−0.0045 (16)	0.0051 (16)	0.0017 (16)
C25	0.0466 (19)	0.0363 (16)	0.0411 (16)	−0.0015 (14)	0.0054 (14)	0.0038 (13)
C26	0.0416 (18)	0.0440 (18)	0.0418 (17)	0.0010 (14)	0.0059 (14)	−0.0029 (14)
C27	0.055 (2)	0.0431 (18)	0.0416 (17)	0.0042 (15)	0.0021 (15)	−0.0021 (15)
C28	0.078 (3)	0.051 (2)	0.057 (2)	0.0031 (19)	0.0170 (19)	−0.0005 (17)
C29	0.110 (3)	0.046 (2)	0.061 (2)	0.006 (2)	0.008 (2)	0.0046 (18)
C30	0.113 (4)	0.055 (2)	0.058 (2)	0.033 (2)	0.003 (2)	−0.0008 (19)
C31	0.077 (3)	0.072 (3)	0.056 (2)	0.029 (2)	0.0095 (19)	0.002 (2)
C32	0.053 (2)	0.055 (2)	0.0449 (18)	0.0139 (17)	0.0031 (15)	−0.0025 (16)
C33	0.0410 (19)	0.067 (2)	0.0424 (17)	0.0075 (17)	0.0030 (14)	−0.0042 (16)
C34	0.044 (2)	0.102 (3)	0.058 (2)	0.012 (2)	0.0087 (17)	0.001 (2)
C35	0.052 (2)	0.117 (4)	0.052 (2)	−0.015 (2)	0.0106 (18)	0.007 (2)
C36	0.067 (2)	0.083 (3)	0.051 (2)	−0.018 (2)	0.0041 (19)	0.0135 (19)
C37	0.056 (2)	0.055 (2)	0.053 (2)	−0.0053 (17)	0.0055 (17)	0.0032 (16)
C38	0.0438 (18)	0.052 (2)	0.0393 (17)	−0.0020 (15)	0.0046 (14)	−0.0004 (15)
C39	0.072 (3)	0.086 (3)	0.099 (3)	0.003 (2)	−0.003 (2)	0.013 (3)
C40	0.086 (3)	0.062 (3)	0.057 (2)	−0.009 (2)	−0.004 (2)	0.015 (2)
C41	0.072 (3)	0.103 (4)	0.100 (3)	−0.019 (3)	0.027 (3)	0.007 (3)
C42	0.071 (3)	0.095 (3)	0.130 (4)	0.000 (3)	0.024 (3)	−0.001 (3)
O3	0.119 (2)	0.086 (2)	0.091 (2)	−0.0147 (19)	0.0023 (18)	0.0400 (18)
O4	0.0705 (18)	0.0692 (18)	0.101 (2)	−0.0052 (14)	0.0190 (15)	0.0229 (15)

### Geometric parameters (Å, °)

**Table:**

O1—C13	1.423 (3)	C22—C23	1.372 (5)
O1—H2	0.93 (4)	C22—H22A	0.9300
O2—C26	1.442 (3)	C23—C24	1.368 (4)
O2—H1	0.88 (4)	C23—H23A	0.9300
C1—C2	1.375 (5)	C24—C25	1.399 (4)
C1—C6	1.394 (4)	C24—H24A	0.9300
C1—C13	1.524 (4)	C25—C26	1.529 (4)
C2—C3	1.394 (5)	C26—C27	1.528 (4)
C2—H2A	0.9300	C26—C38	1.532 (4)
C3—C4	1.369 (6)	C27—C28	1.372 (4)
C3—H3A	0.9300	C27—C32	1.396 (4)
C4—C5	1.373 (6)	C28—C29	1.391 (5)
C4—H4A	0.9300	C28—H28A	0.9300
C5—C6	1.388 (5)	C29—C30	1.372 (5)
C5—H5A	0.9300	C29—H29A	0.9300
C6—C7	1.457 (5)	C30—C31	1.370 (5)
C7—C12	1.382 (5)	C30—H30A	0.9300
C7—C8	1.397 (5)	C31—C32	1.384 (4)
C8—C9	1.370 (6)	C31—H31A	0.9300
C8—H8A	0.9300	C32—C33	1.464 (4)
C9—C10	1.375 (7)	C33—C38	1.386 (4)
C9—H9A	0.9300	C33—C34	1.390 (4)
C10—C11	1.394 (5)	C34—C35	1.378 (5)
C10—H10A	0.9300	C34—H34A	0.9300
C11—C12	1.385 (5)	C35—C36	1.374 (5)
C11—H11A	0.9300	C35—H35A	0.9300
C12—C13	1.537 (4)	C36—C37	1.386 (5)
C13—C14	1.534 (4)	C36—H36A	0.9300
C14—C15	1.396 (4)	C37—C38	1.379 (4)
C14—C19	1.404 (4)	C37—H37A	0.9300
C15—C16	1.378 (4)	C39—C40	1.478 (5)
C15—H15A	0.9300	C39—H39A	0.9600
C16—C17	1.372 (5)	C39—H39B	0.9600
C16—H16A	0.9300	C39—H39C	0.9600
C17—C18	1.377 (4)	C40—O3	1.197 (4)
C17—H17A	0.9300	C40—O4	1.323 (4)
C18—C19	1.391 (4)	C41—O4	1.454 (4)
C18—H18A	0.9300	C41—C42	1.491 (5)
C19—C20	1.514 (4)	C41—H41A	0.9700
C20—C21	1.393 (4)	C41—H41B	0.9700
C20—C25	1.403 (4)	C42—H42A	0.9600
C21—C22	1.383 (4)	C42—H42B	0.9600
C21—H21A	0.9300	C42—H42C	0.9600
			
C13—O1—H2	106 (2)	C24—C23—H23A	120.0
C26—O2—H1	106 (3)	C22—C23—H23A	120.0
C2—C1—C6	120.9 (3)	C23—C24—C25	122.5 (3)
C2—C1—C13	128.0 (3)	C23—C24—H24A	118.7
C6—C1—C13	111.1 (3)	C25—C24—H24A	118.7
C1—C2—C3	118.5 (4)	C24—C25—C20	118.2 (3)
C1—C2—H2A	120.8	C24—C25—C26	115.7 (3)
C3—C2—H2A	120.8	C20—C25—C26	126.1 (3)
C4—C3—C2	120.6 (5)	O2—C26—C27	110.9 (2)
C4—C3—H3A	119.7	O2—C26—C25	108.4 (2)
C2—C3—H3A	119.7	C27—C26—C25	112.5 (2)
C3—C4—C5	121.3 (4)	O2—C26—C38	110.0 (2)
C3—C4—H4A	119.4	C27—C26—C38	101.5 (2)
C5—C4—H4A	119.4	C25—C26—C38	113.4 (2)
C4—C5—C6	118.9 (4)	C28—C27—C32	120.5 (3)
C4—C5—H5A	120.6	C28—C27—C26	129.3 (3)
C6—C5—H5A	120.6	C32—C27—C26	110.2 (3)
C5—C6—C1	119.9 (4)	C27—C28—C29	118.6 (3)
C5—C6—C7	131.5 (4)	C27—C28—H28A	120.7
C1—C6—C7	108.6 (3)	C29—C28—H28A	120.7
C12—C7—C8	119.6 (4)	C30—C29—C28	120.5 (4)
C12—C7—C6	109.0 (3)	C30—C29—H29A	119.7
C8—C7—C6	131.4 (4)	C28—C29—H29A	119.7
C9—C8—C7	118.7 (5)	C31—C30—C29	121.4 (3)
C9—C8—H8A	120.6	C31—C30—H30A	119.3
C7—C8—H8A	120.6	C29—C30—H30A	119.3
C8—C9—C10	121.7 (5)	C30—C31—C32	118.5 (3)
C8—C9—H9A	119.2	C30—C31—H31A	120.7
C10—C9—H9A	119.2	C32—C31—H31A	120.7
C9—C10—C11	120.3 (5)	C31—C32—C27	120.4 (3)
C9—C10—H10A	119.8	C31—C32—C33	130.7 (3)
C11—C10—H10A	119.8	C27—C32—C33	108.8 (3)
C12—C11—C10	118.0 (4)	C38—C33—C34	119.7 (3)
C12—C11—H11A	121.0	C38—C33—C32	109.1 (3)
C10—C11—H11A	121.0	C34—C33—C32	131.1 (3)
C7—C12—C11	121.6 (3)	C35—C34—C33	118.9 (3)
C7—C12—C13	111.0 (3)	C35—C34—H34A	120.6
C11—C12—C13	127.4 (3)	C33—C34—H34A	120.6
O1—C13—C1	107.5 (2)	C36—C35—C34	121.1 (3)
O1—C13—C14	112.8 (2)	C36—C35—H35A	119.4
C1—C13—C14	112.6 (2)	C34—C35—H35A	119.4
O1—C13—C12	110.2 (2)	C35—C36—C37	120.5 (4)
C1—C13—C12	100.4 (3)	C35—C36—H36A	119.7
C14—C13—C12	112.7 (3)	C37—C36—H36A	119.7
C15—C14—C19	118.1 (3)	C38—C37—C36	118.5 (3)
C15—C14—C13	117.2 (3)	C38—C37—H37A	120.7
C19—C14—C13	124.8 (3)	C36—C37—H37A	120.7
C16—C15—C14	122.4 (3)	C37—C38—C33	121.2 (3)
C16—C15—H15A	118.8	C37—C38—C26	128.4 (3)
C14—C15—H15A	118.8	C33—C38—C26	110.3 (3)
C17—C16—C15	119.4 (3)	C40—C39—H39A	109.5
C17—C16—H16A	120.3	C40—C39—H39B	109.5
C15—C16—H16A	120.3	H39A—C39—H39B	109.5
C16—C17—C18	119.2 (3)	C40—C39—H39C	109.5
C16—C17—H17A	120.4	H39A—C39—H39C	109.5
C18—C17—H17A	120.4	H39B—C39—H39C	109.5
C17—C18—C19	122.7 (3)	O3—C40—O4	122.3 (4)
C17—C18—H18A	118.7	O3—C40—C39	125.3 (4)
C19—C18—H18A	118.7	O4—C40—C39	112.4 (3)
C18—C19—C14	118.3 (3)	O4—C41—C42	107.5 (3)
C18—C19—C20	114.4 (3)	O4—C41—H41A	110.2
C14—C19—C20	126.7 (3)	C42—C41—H41A	110.2
C21—C20—C25	117.8 (3)	O4—C41—H41B	110.2
C21—C20—C19	114.2 (3)	C42—C41—H41B	110.2
C25—C20—C19	127.6 (3)	H41A—C41—H41B	108.5
C22—C21—C20	123.2 (3)	C41—C42—H42A	109.5
C22—C21—H21A	118.4	C41—C42—H42B	109.5
C20—C21—H21A	118.4	H42A—C42—H42B	109.5
C23—C22—C21	118.4 (3)	C41—C42—H42C	109.5
C23—C22—H22A	120.8	H42A—C42—H42C	109.5
C21—C22—H22A	120.8	H42B—C42—H42C	109.5
C24—C23—C22	120.0 (3)	C40—O4—C41	117.0 (3)

### Hydrogen-bond geometry (Å, °)

**Table:**

*D*—H···*A*	*D*—H	H···*A*	*D*···*A*	*D*—H···*A*
O2—H1···O3^i^	0.88 (4)	1.90 (4)	2.779 (3)	173 (4)
O1—H2···O2	0.93 (4)	1.84 (4)	2.739 (3)	161 (3)

Symmetry codes: (i) −*x*+1, *y*+1/2, −*z*+1/2.
